# Characterizing Spatial Dynamics of Bifurcation to Alternans in Isolated Whole Rabbit Hearts Based on Alternate Pacing

**DOI:** 10.1155/2015/170768

**Published:** 2015-10-25

**Authors:** Kanchan Kulkarni, Ramjay Visweswaran, Xiaopeng Zhao, Elena G. Tolkacheva

**Affiliations:** ^1^Department of Biomedical Engineering, University of Minnesota, 312 Church Street SE, Minneapolis, MN 55455, USA; ^2^Department of Mechanical, Aerospace, and Biomedical Engineering, University of Tennessee, 1506 Middle Drive, Knoxville, TN 37996, USA

## Abstract

Sudden cardiac death instigated by ventricular fibrillation (VF) is the largest cause of natural death in the USA. Alternans, a beat-to-beat alternation in the action potential duration, has been implicated as being proarrhythmic. The onset of alternans is mediated via a bifurcation, which may occur through either a smooth or a border-collision mechanism. The objective of this study was to characterize the mechanism of bifurcation to alternans based on experiments in isolated whole rabbit hearts. High resolution optical mapping was performed and the electrical activity was recorded from the left ventricle (LV) epicardial surface of the heart. Each heart was paced using an “alternate pacing protocol,” where the basic cycle length (BCL) was alternatively perturbed by ±*δ*. Local onset of alternans in the heart, BCL_start_, was measured in the absence of perturbations (*δ* = 0) and was defined as the BCL at which 10% of LV exhibited alternans. The influences of perturbation size were investigated at two BCLs: one prior to BCL_start_ (BCL_prior_ = BCL_start_ + 20 ms) and one preceding BCL_prior_ (BCL_far_ = BCL_start_ + 40 ms). Our results demonstrate significant spatial correlation of the region exhibiting alternans with smooth bifurcation characteristics, indicating that transition to alternans in isolated rabbit hearts occurs predominantly through smooth bifurcation.

## 1. Introduction

Ventricular fibrillation (VF), manifesting as chaotic unsynchronized electrical activity in the heart, is known to cause sudden cardiac death (SCD). SCD is one of the largest natural causes of death, killing more than 300,000 people annually in the United States [1, 2]. Alternans, which is a beat-to-beat alternation in the action potential duration (APD), has been implicated as being proarrhythmic and a potential source of cardiac instability [3–6]. When the heart is paced at progressively increasing rates, electrical restitution, which is a fundamental property of cardiac myocytes, causes the heart to bifurcate from a constant APD response at lower frequencies to an alternating long-short APD pattern (alternans) at higher frequencies [7–11]. Characterizing this bifurcation can provide useful insights into understanding the dynamics of the cardiac system. In particular, knowledge of the bifurcation type that governs the transition to alternans can potentially be used as a means to predict the formation of alternans prior to their onset [12], which may be useful in preventing arrhythmias.

Previous research has implicated both smooth and border-collision bifurcations when trying to characterize the transition to alternans in small cardiac tissue. Initially, Nolasco and Dahlen modeled transition to alternans as a smooth bifurcation [7]; however, later studies reported a border-collision type of bifurcation to alternans [13]. More recently, it was shown that a more complex bifurcation model involving the coexistence of both smooth and border-collision characteristics may exist in the heart. Based on experiments on adult bullfrog ventricular tissue samples, Berger et al. suggested the existence of a smooth-like behavior close to the bifurcation point and a border-collision type behavior further away from the bifurcation point [14]. Although the presence of different bifurcation characteristics in small tissue samples suggests very interesting dynamical behavior, their study was limited by the use of glass microelectrode recordings taken at single point locations. Therefore, the spatial distribution of the bifurcation characteristics and their correlation to alternans remains to be validated.

The main objective of this study was to experimentally investigate the spatial dynamics of bifurcations that governs the transition to alternans in isolated whole rabbit hearts. For this purpose, we applied an* “alternate pacing protocol,”* originally introduced by Heldstab et al. [15] and further experimentally investigated by Berger et al. [14]. Theoretical studies by Zhao et al. [16, 17] show that smooth and border-collision bifurcations to alternans demonstrate qualitatively different trends in their response to this alternate pacing protocol. We aimed to use this protocol to characterize the type of bifurcation to alternans and study its spatial distribution across the left ventricular (LV) surface of the heart. We also aimed to investigate the spatial correlation between the regions of the heart exhibiting alternans and the different bifurcation characteristics.

## 2. Methods

### 2.1. Optical Mapping

All experiments were performed in accordance with the guidelines of the Institutional Animal Care and Use Committee at the University of Minnesota. New Zealand White rabbits (Bakkom Rabbitry, 1.3–2.0 kg, *n* = 4) were injected with heparin sulfate (550 U/100 g) and anesthetized with ketamine and xylazine (35 mg/kg and 5 mg/kg, resp.), as described previously [18, 19]. After a thoracotomy was performed, the heart was quickly removed and immersed in cardioplegic solution (in mM: glucose 280, KCl 13.44, NaHCO_3_ 12.6, and mannitol 34). The aorta was quickly cannulated and retrogradely perfused with warm (37 ± 1°C) oxygenated Tyrode's solution (in mM: NaCl 130, CaCl_2_ 1.8, KCl 4, MgCl_2_ 1.0, NaH_2_PO_4_ 1.2, NaHCO_3_ 24, glucose 5.5, and pH 7.4) under constant pressure. The heart was immersed in a chamber and superfused with the same Tyrode's solution. Blebbistatin (10 *μ*M) was added to Tyrode's solution to reduce motion artifacts.

A bolus of 4 mL of the voltage-sensitive dye di-4-ANEPPS (10 *μ*M) was injected and excited with the use of a diode-pumped, continuous-excitation green laser (532 nm, 1 W; Shanghai Dream Lasers Technology, Shanghai, China). Optical movies corresponding to the fluorescence signal were recorded from the epicardial surface of the LV by fast (1000 frames per second) 14-bit resolution, 80 × 80-pixel resolution camera (Little Joe, RedShirt Imaging, SciMeasure) after a period of stabilization (~30 minutes).

### 2.2. Alternate Pacing Protocol

**Figure 1:**
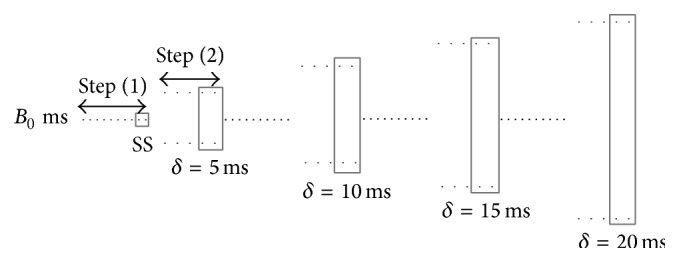
Schematic of the alternate pacing protocol for a specific BCL *B*_0_. Grey boxes indicate responses that have been captured during optical mapping experiments. Local onset of alternans was calculated based on steady state (SS) responses at *δ* = 0, while alternans induced by perturbations was calculated based on responses captured at increasing *δ* values.

External stimuli (5 ms duration, twice the threshold) were applied to the base of the LV surface of the isolated Langendorff-perfused rabbit hearts and the following alternate pacing protocol [14] was applied (see Figure 1).

(1) Forty stimuli at a constant BCL (denoted by *B*_0_) value were applied to achieve steady state (SS).
(2) Forty alternating stimuli were applied at BCL_*n*_ = *B*_0_ + (−1)^*n*^ *δ*, where *δ* = 5 ms is the perturbation amplitude.
(3) Steps (1) and (2) were repeated for *δ* values of 10, 15, and 20 ms, respectively.

The baseline BCL *B*_0_ was progressively reduced from 300 ms down to 160 ms in steps of 20 ms. Optical movies were acquired to capture the responses to last ten stimuli during Steps (1) and (2) for each BCL. The alternate pacing protocol was repeated twice for each heart, so a total of 8 protocols were analyzed across all hearts.

### 2.3. Data Analysis

**Figure 2:**
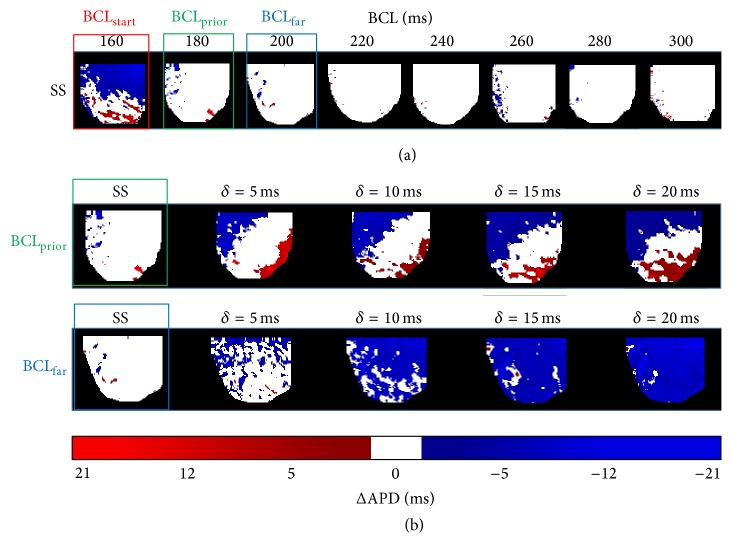
(a) Representative 2D alternans maps for SS BCLs showing the local onset of alternans at BCL_start_ (red box). Two prior BCLs, BCL_prior_ and BCL_far,_ are also shown (green and blue boxes). (b) 2D alternans maps induced by perturbations *δ* at BCL_prior_ and BCL_far_. Color bar represents the amplitude of alternans.

At each baseline BCL *B*_0_, APD was measured at 80% repolarization for each pixel. The APD responses for the last ten stimuli captured were divided in pairs and the amplitude of alternans was calculated as the absolute difference between even and odd APD responses: ΔAPD = |APD_even_ − APD_odd_|. The average amplitude of alternans was calculated across all five pairs, and the temporal threshold for APD alternans was set at 5 ms. Two-dimensional (2D) alternans maps corresponding to SS responses (see Figure 2) were generated for the LV surface for each baseline BCL and were used to identify the local spatial onset of alternans (BCL_start_) in the heart as described previously [18]. Specifically, BCL_start_ was identified as the SS *B*_0_ at which at least 10% of the LV surface exhibited alternans. The baseline BCL *B*_0_ just prior to BCL_start_ was denoted by BCL_prior_, and the BCL preceding BCL_prior_ was denoted by BCL_far_. Furthermore, the alternans was identified as either spatially discordant or spatially concordant based on the presence or absence of alternans with opposite phases, respectively [20]. Alternans was considered as spatially discordant if at least 5% of the alternans exhibited had opposite phases.  Table 1 shows the total number of either type of steady state alternans for BCL_start_, as well as alternans induced by *δ* = 5 ms perturbations for BCL_prior_ and BCL_far_.

At each pixel, an amplification Gain was calculated as described previously [14]:

(1) $$\begin{array}{c} \text{Gain}=\frac{\left|\Delta \text{APD}\right|}{2\delta }. \end{array}$$

Theoretical investigations in [16] demonstrate that the Gain versus *δ* relation exhibits a decreasing trend as *δ* increases for smooth bifurcation, whereas the Gain versus *δ* relation exhibits an increasing trend as *δ* increases for border-collision bifurcation. At each pixel, Gain was calculated for each *δ* at BCL_prior_ and BCL_far_, respectively. Then, the dependence of Gain on *δ* was fitted using a linear curve at BCL_prior_ and BCL_far_, respectively. The correlation coefficient* R*-square was then calculated to evaluate the quality of fitting. For* R*-square > 0.4, a decrease or increase in Gain with *δ* was identified as smooth or border-collision bifurcation, respectively. To account for experimental noise, a threshold of 0.07 (or smaller) between consecutive Gain values was considered acceptable while determining the trend. The bifurcation was considered as undetermined if the* R*-square < 0.4 or if the consecutive Gain difference > 0.07.

### 2.4. Statistics

All data are presented as mean ± standard error. Statistical comparisons between the three bifurcation types were performed using ANOVA (Origin Software, Northampton, MA, USA). Values of *P* < 0.05 were considered statistically significant.

## 3. Results

### 3.1. Spatial Distribution of Bifurcation Characteristics

First, we studied the local onset of SS alternans (Step (1) of alternate pacing protocol) and alternans that are induced by small perturbations *δ* (Step (2) of the alternate pacing protocol). Figure 2(a) shows a representative example of the 2D alternans maps generated for different baseline BCLs *B*_0_ at SS. Here, blue and red regions indicate the presence of spatially discordant alternans (see color bar for alternans phases) while the white regions indicate the absence of alternans. In this example, the local onset of alternans, BCL_start_ (red box), occurred at 160 ms since more than 10% of the LV was occupied by alternans at this BCL. Consequently, BCL_prior_ and BCL_far_ (green and blue boxes) were identified as 180 ms and 200 ms, respectively. Figure 2(b) shows 2D maps illustrating the development of alternans induced by small perturbations *δ* that were applied at BCL_prior_ and BCL_far_. Note that alternans is spatially discordant for BCL_prior_ and spatially concordant for BCL_far_. At both BCL_prior_ and BCL_far_, we observed that the heart was more prone to the formation of alternans as the perturbation size *δ* was increased, which is indicated by the progressive increase in the red and blue color as *δ* increases.

**Figure 3:**
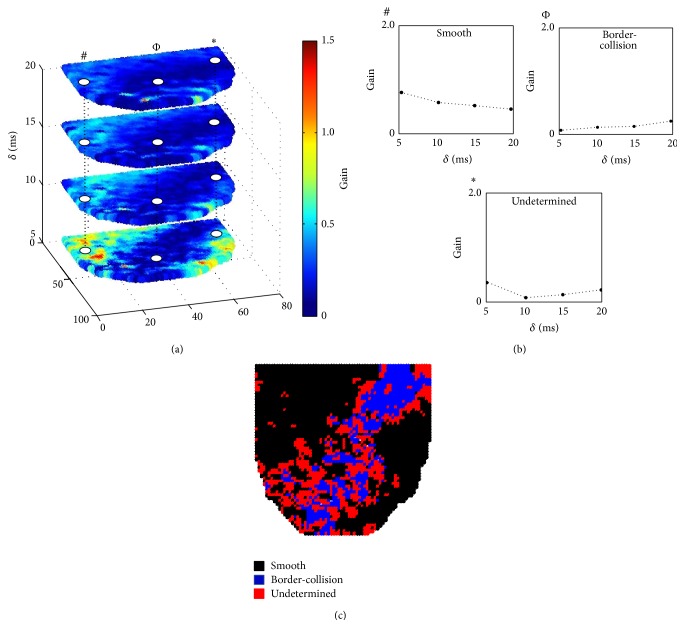
(a) Representative 2D Gain maps as a function of *δ* calculated at BCL_prior_ for example shown in Figure 2(b). (b) Single-pixel trends in Gain as a function of *δ* for the three representative pixels marked in panel (a). Note that # suggests the presence of smooth bifurcation, and Φ suggests the presence of border-collision bifurcation. However, the type of bifurcation in pixel ∗ cannot be identified. (c) 2D bifurcation map showing the spatial distribution of different bifurcations for panel (a).

It has been previously demonstrated, using numerical simulations [16], that bifurcation to alternans can be identified by looking at the changes in the amplification Gain as a function of *δ*. Therefore, for each *δ* we constructed 2D Gain maps to investigate the spatial distribution of the changes in Gain.  Figure 3(a) shows a representative example of 2D Gain maps that were generated for BCL_prior_ from Figure 2(b), showing the spatial distribution of Gain for various *δ*.  Figure 3(b) illustrates three representative single-pixel examples from Figure 3(a) (#, Φ, and ∗) indicating the presence of different trends in Gain as a function of *δ*. It has been shown previously that a decrease in Gain with increase in *δ* indicates the presence of smooth bifurcation (see pixel # in Figure 3(b)), while an increase in Gain with an increase in *δ* characterizes border-collision bifurcation (see pixel Φ in Figure 3(b)) [14, 16]. We also found that at some pixels the type of bifurcation could not be determined (see pixel ∗ in Figure 3(b)).

Figure 3(c) shows a representative 2D bifurcation map demonstrating the spatial distribution of different bifurcations for panel (a). Here, the region in black corresponds to the area of the heart (60.7%) that went to alternans through smooth bifurcation, the region in blue indicates border-collision bifurcation (16.8%), and bifurcation type could not be determined for the region in red (22.5%). Therefore, our analysis suggests that the heart transitions to alternans predominantly through smooth bifurcation.

**Figure 4:**
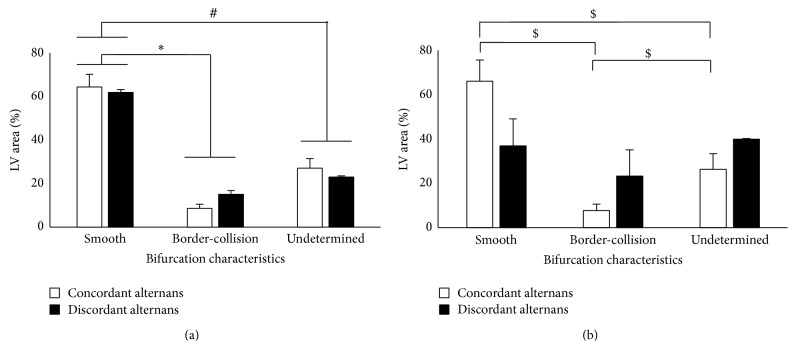
Mean bifurcation characteristics (in % of LV surface) in all experiments calculated for spatially concordant and discordant alternans at (a) BCL_prior_ and (b) BCL_far_. ∗ indicates significance of *P* < 0.05 between smooth and border-collision bifurcations. # indicates significance of *P* < 0.05 between smooth and undetermined bifurcations. $ indicates significance of *P* < 0.05 between bifurcation types for concordant alternans.

Finally, we quantified the percentage of LV area of the heart with smooth, border-collision and undetermined bifurcations.  Figure 4 shows the average percentage of LV area for each type of bifurcation separately for BCL_prior_ (Figure 4(a)) and BCL_far_ (Figure 4(b)) calculated across all our experiments. As seen from Figure 4, at BCL_prior_, for both spatially concordant and discordant alternans, the percentage of LV area of the heart that exhibited smooth bifurcation was significantly larger in comparison to the one with border-collision and undetermined bifurcations. At BCL_far_, this result was valid for the case of spatially concordant alternans but not for the spatially discordant alternans. Our results suggest that, just prior to the onset of alternans, we predominantly observe smooth bifurcation characteristics in the heart, irrespective of the spatial pattern of alternans.

### 3.2. Spatial Correlation of Local Onset of Alternans with Bifurcation Characteristics

We also investigated the spatial correlation of the regions exhibiting alternans with the different bifurcation characteristics.

**Figure 5:**
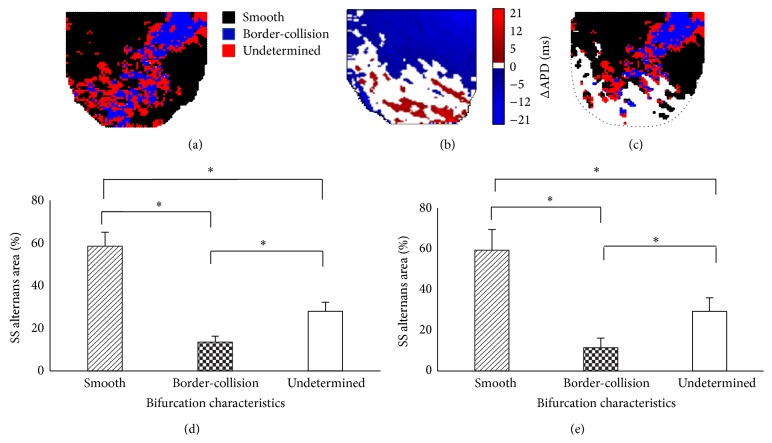
Spatial correlation of the local onset of SS alternans with bifurcation characteristics. (a) 2D bifurcation map calculated at BCL_prior_ (see Figure 3(c)). (b) 2D SS alternans map calculated at BCL_start_ (see Figure 2(a)). (c) Correlation between bifurcation and SS alternans maps. Average % of smooth, border-collision, and undetermined bifurcations from all experiments are shown for (d) BCL_prior_ and (e) BCL_far_. ∗ indicates *P* < 0.05.

First, we studied the spatial correlation between the regions of SS alternans and the bifurcation characteristics. Figures 5(a) and 5(b) show representative examples of 2D bifurcation map generated at BCL_prior_ (see Figure 3(c)) and the SS alternans map generated at BCL_start_ (see Figure 2(a)), respectively. The spatial superposition of these two maps is shown in Figure 5(c). Therefore, in Figure 5(c) the bifurcation characteristics (calculated at BCL_prior_) are only shown for spatial regions that exhibited alternans at the next BCL (i.e., BCL_start_). The white regions in Figures 5(b) and 5(c) were excluded from the analysis, since no alternans was present there at BCL_start_. Figures 5(d) and 5(e) show the average percentage of LV area that developed SS alternans with the bifurcation characteristics calculated across all our experiments at BCL_prior_ and BCL_far_, respectively. Since the spatial pattern of alternans developed at BCL_start_ did not always coincide with the spatial pattern of alternans induced by perturbation, it was difficult to categorize the bifurcation characteristics in relation to spatially concordant or discordant alternans. However, as seen from the figure, the overall regions of SS alternans that coincided with smooth bifurcation characteristics were significantly higher than those coinciding with border-collision bifurcation, both at BCL_prior_ and BCL_far_. The data suggests that the regions of the LV that eventually develop alternans at SS show predominantly smooth bifurcation characteristics prior to the onset of alternans.

**Figure 6:**
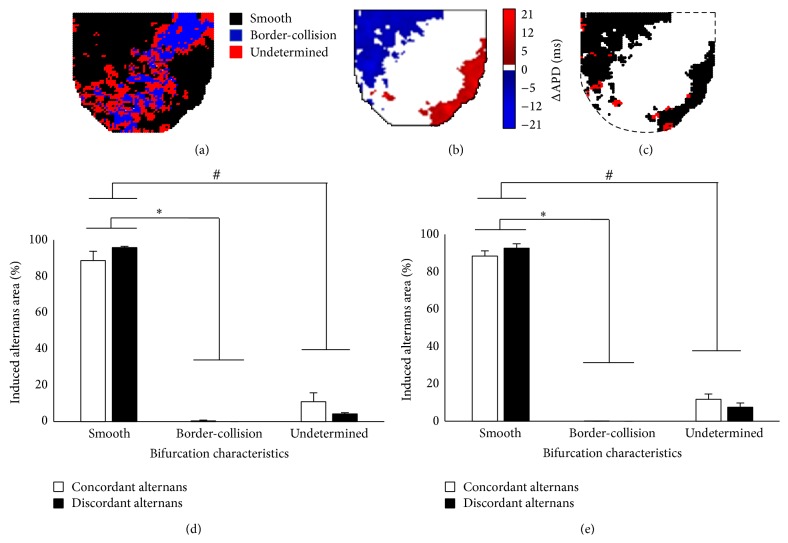
Spatial correlation of alternans induced by perturbation (at *δ* = 5 ms) with bifurcation characteristics. (a) 2D bifurcation map calculated at BCL_prior_ (see Figure 3(c)). (b) 2D alternans map at *δ* = 5 ms calculated at BCL_prior_ (see Figure 2(b)). (c) Spatial correlation between these two maps. Average % of smooth, border-collision, and undetermined bifurcations from all experiments are shown for (d) BCL_prior_ and (e) BCL_far_. ∗ indicates significance of *P* < 0.05 between smooth and border-collision bifurcations. # indicates significance of *P* < 0.05 between smooth and undetermined bifurcations.

Similar analysis was performed to investigate the spatial correlation between bifurcation characteristics and alternans induced by alternate pacing. Figures 6(a) and 6(b) show a representative example of 2D bifurcation map (see Figure 3(c)) and 2D alternans map at *δ* = 5 ms (see Figure 2(b)), respectively. Note that both maps were calculated at BCL_prior_.  Figure 6(c) shows the spatial correlation between these two maps. Figures 6(d) and 6(e) show the average data across all experiments for BCL_prior_ and BCL_far_, for both spatially concordant and discordant alternans. As seen from the figure, the region of induced alternans coincided predominantly with smooth bifurcation characteristics, both at BCL_prior_ and BCL_far_, irrespective of the type of alternans. Note that the percentage of border-collision bifurcation is negligible. The data suggests that alternans that is induced by small perturbations is formed predominantly through smooth bifurcation.

Comparison of data across Figures 5 and 6 suggests a higher spatial correlation between smooth bifurcation characteristics and alternans induced by perturbation as opposed to SS alternans. The induced alternans showed lower border-collision and undetermined characteristics than SS alternans at both BCL_prior_ and BCL_far_.

## 4. Discussion

Characterizing the type of bifurcation to alternans holds promise as a possible method for the prediction of alternans prior to its onset and may shed light for the prevention of arrhythmias. In this study, we experimentally investigated the spatial dynamics of bifurcation that governs the transition to alternans in isolated whole rabbit hearts. We identified the local onset of alternans, characterized the bifurcation type prior to its onset, and studied the spatial distribution of bifurcation characteristics across the LV surface of the heart. In addition, we also investigated the spatial correlation of the regions of the LV exhibiting alternans with the type of bifurcation. To the authors' best knowledge, this is the first study to investigate the bifurcation type of alternans based on spatial dynamics and quantify the spatial correlation of alternans with the type of bifurcation exhibited.

Our main results are as follows. First, the bifurcation to alternans in the heart occurs predominantly through smooth bifurcation. Second, the region of the heart eventually exhibiting alternans shows a significantly higher spatial correlation to the region exhibiting smooth bifurcation characteristics.

Previous studies attempted to characterize the bifurcation to alternans based on theoretical models of the atrioventricular nodes [13] or microelectrode recordings from ventricular tissue samples [14]. Here, we aimed to investigate the spatial distribution of the bifurcation characteristics by performing high resolution optical mapping experiments on isolated whole rabbit hearts. Although initially described as a period doubling smooth bifurcation, later studies of alternans showed the existence of border-collision bifurcation characteristics and a more complex behavior that governed the transition to alternans. Our results indicated a spatial predominance to smooth bifurcation when transitioning from constant APD response to alternans in the LV region of the heart. Although the presence of border-collision bifurcation was seen, the region of the LV exhibiting smooth bifurcation was significantly higher.

We also investigated the spatial correlation of the regions of the LV exhibiting alternans with the type of bifurcation in the case of both spatially concordant and spatially discordant alternans. As was seen from the results, there was significantly higher spatial correlation between alternans induced by perturbation with smooth bifurcation characteristics. For the purpose of this study, we only considered alternans induced by a perturbation of 5 ms when investigating the spatial correlation between the induced alternans and the type of bifurcation. Since, as shown in the results, increasing the perturbation resulted in an increase in the area of the LV exhibiting alternans, a perturbation of 5 ms was a good representation of the onset of induced alternans. An interesting finding was that the regions of the LV that exhibited alternans at SS did not exactly coincide with the regions of the LV in which alternans was induced by perturbation. The phenomenon of spatial concordance or discordance of alternans also differed between SS and induced alternans. This can be attributed to the possibility that the alternate pacing protocol leads to altered dynamical activity of the heart compared to SS pacing. However, in both cases irrespective of the spatial pattern of alternans, just prior to the onset we see a significantly higher spatial correlation of alternans with smooth bifurcation characteristics as compared with either the border-collision bifurcation or the undetermined bifurcation characteristics.

Our results indicated that the region of the LV exhibiting border-collision bifurcation was significantly low while a definite portion of the LV exhibited undetermined characteristics. This indicates the possibility of the presence of other bifurcation characteristics in the heart with more complex behavior. However, in comparison to smooth bifurcation, the region with undetermined characteristics was still significantly smaller just prior to the onset of alternans, which supports the result that the transition to alternans predominantly occurs through a smooth bifurcation.

## 5. Conclusion

We observed that the transition from constant APD response to alternans in isolated whole rabbit hearts occurred primarily through a smooth bifurcation. There was significant spatial correlation of the region exhibiting alternans with smooth bifurcation characteristics. The correlation of smooth bifurcation with alternans induced by perturbation was significantly higher than with SS alternans. As we moved away from the onset of alternans, the percentage of the heart exhibiting smooth bifurcation characteristics decreased as the region with undetermined bifurcation characteristics increased. The region of the heart exhibiting border-collision bifurcation characteristics was significantly smaller than both the smooth bifurcation and the undetermined bifurcation regions.

## Figures and Tables

**Table 1:** Classification of spatial pattern of alternans as concordant or discordant alternans for BCL_start_, BCL_prior_, and BCL_far_ for all experiments.

BCL	Total protocols (4 rabbits, 8 protocols total)	# of spatially concordant alternans	# of spatially discordant alternans
BCL_start_	8	3	5
BCL_prior_ (*δ* = 5 ms)	8	6	2
BCL_far_ (*δ* = 5 ms)	8	6	2

## References

[1] George A. L. (2013). Molecular and genetic basis of sudden cardiac death. *The Journal of Clinical Investigation*.

[2] Zipes D. P., Wellens H. J. J. (1998). Sudden cardiac death. *Circulation*.

[3] Karma A. (1994). Electrical alternans and spiral wave breakup in cardiac tissue. *Chaos*.

[4] Gilmour R. F. (2002). Electrical restitution and ventricular fibrillation: negotiating a slippery slope. *Journal of Cardiovascular Electrophysiology*.

[5] Watanabe M., Otani N. F., Gilmour R. F. (1995). Biphasic restitution of action potential duration and complex dynamics in ventricular myocardium. *Circulation Research*.

[6] Fox J. J., McHarg J. L., Gilmour R. F. (2002). Ionic mechanism of electrical alternans. *The American Journal of Physiology—Heart and Circulatory Physiology*.

[7] Nolasco J. B., Dahlen R. W. (1968). A graphic method for the study of alternation in cardiac action potentials. *Journal of Applied Physiology*.

[8] Tolkacheva E. G., Schaeffer D. G., Gauthier D. J., Krassowska W. (2003). Condition for alternans and stability of the 1:1 response pattern in a ‘memory’ model of paced cardiac dynamics. *Physical Review E: Statistical, Nonlinear, and Soft Matter Physics*.

[9] Riccio M. L., Koller M. L., Gilmour R. F. (1999). Electrical restitution and spatiotemporal organization during ventricular fibrillation. *Circulation Research*.

[10] Koller M. L., Riccio M. L., Gilmour R. F. (1998). Dynamic restitution of action potential duration during electrical alternans and ventricular fibrillation. *The American Journal of Physiology—Heart and Circulatory Physiology*.

[11] Hastings H. M., Fenton F. H., Evans S. J. (2000). Alternans and the onset of ventricular fibrillation. *Physical Review E*.

[12] Petrie A., Zhao X. (2012). Estimating eigenvalues of dynamical systems from time series with applications to predicting cardiac alternans. *Proceedings of The Royal Society of London, Series A: Mathematical, Physical and Engineering Sciences*.

[13] Sun J., Amellal F., Glass L., Billette J. (1995). Alternans and period-doubling bifurcations in atrioventricular nodal conduction. *Journal of Theoretical Biology*.

[14] Berger C. M., Zhao X., Schaeffer D. G., Dobrovolny H. M., Krassowska W., Gauthier D. J. (2007). Period-doubling bifurcation to alternans in paced cardiac tissue: crossover from smooth to border-collision characteristics. *Physical Review Letters*.

[15] Heldstab J., Thomas H., Geisel T., Radons G. (1983). Linear and nonlinear response of discrete dynamical systems. I. Periodic attractors. *Zeitschrift für Physik B Condensed Matter*.

[16] Zhao X., Schaeffer D. G. (2007). Alternate pacing of border-collision period-doubling bifurcations. *Nonlinear Dynamics*.

[17] Zhao X., Schaeffer D. G., Berger C. M., Gauthier D. J., Krassowska W. (2008). Cardiac alternans arising from an unfolded border-collision bifurcation. *Journal of Computational and Nonlinear Dynamics*.

[18] Smith R. M., Visweswaran R., Talkachova I., Wothe J. K., Tolkacheva E. G. (2013). Uncoupling the mitochondria facilitates alternans formation in the isolated rabbit heart. *American Journal of Physiology—Heart and Circulatory Physiology*.

[19] Cram A. R., Rao H. M., Tolkacheva E. G. (2011). Toward prediction of the local onset of alternans in the heart. *Biophysical Journal*.

[20] Weiss J. N., Karma A., Shiferaw Y., Chen P.-S., Garfinkel A., Qu Z. (2006). From pulsus to pulseless: the saga of cardiac alternans. *Circulation Research*.

